# Risk perception among residents living near industries in Godawari Municipality of Lalitpur, Nepal

**DOI:** 10.5620/eaht.2023029

**Published:** 2023-12-29

**Authors:** Kshitij Karki, Anish Chaurel, Aashish Kumar Neupane, Keshab Parajuli, Raju Ghimire

**Affiliations:** 1 Department of Public Health, Asian College for Advance Studies, Purbanchal University, Lalitpur, Nepal

**Keywords:** Self reports, health effects, Risk perception, Nepal, Residents, Industries

## Abstract

The industrial sector stands out as a significant contributor to environmental pollution. Those who reside in close proximity to industrial areas commonly harbor concerns about potential health and environmental hazards. This study aimed to find out the perception of risk and self-reported health impacts among individuals living near industries in Godawari Municipality, Lalitpur, Nepal. Conducted as a community-based cross-sectional study, it involved 270 households. Face-to-face interviews were employed, utilizing a pretested structured questionnaire. The study zone encompassed the communities of Godawari Municipality within a 3-kilometer radius of industrial sites. Specifically, stone mines, stone crushers, and brick kilns were purposefully selected, while study participants were randomly sampled using a random table. Data analysis was performed using IBM SPSS, incorporating both univariate and bivariate techniques. Among those residing near industrial zones, a mere 9.6 % reported experiencing wheezing or whistling in the past 12 months. A substantial 36.3% consistently felt stressed due to industrial activities in their vicinity. Approximately half (51.9 %) of the participants indicated that the contaminated air in the area had adverse effects on human health. Furthermore, a palpable perception of elevated risk was associated with the proximity of industries (p<0.001). Over half of the participants perceived a notable risk stemming from the presence of industries near their homes, largely due to pollutants. These individuals also disclosed various health repercussions and expressed significant apprehension regarding their future well-being in the area. The implications of these findings are substantial, particularly for local-level planning and the development of industrial sites. Addressing the concerns surrounding people's heightened perception of risk from nearby industries is pivotal in fostering harmonious coexistence and informed decision-making.

## Introduction

People's perception of their own health can be used to predict future health-care utilization and fatality rates. Age is also associated to self-reported health state, with older persons being more subjective to ill health than younger people [1]. A risk perception is a judgment about the harmful consequences of certain hazards that can be made by an individual, a group of individuals, or a society [2].

Emission control mechanisms are commonly neglected which is a major challenge for developing countries due to inadequate legislation, budgetary constraints, and lack of political will. It is considered a major public health crisis in developing countries [3].

A 2007 World Health Organization (WHO) report revealed that globally up to 13 million deaths could be averted annually through better environmental management and in some countries over 30 % of the disease burden could be prevented. The burden of disease is attributed due to air pollution, which kills over 2 million people prematurely [4].

Industrial areas have been associated not only with industrial pollution but also with a lack of sanitation and environmental hygiene. Studies have shown that the presence of industry in close proximity to residences has disadvantages like wastewater stagnation, cooking problems, occupational dust from brick and cement kilns, occupational gas and irritating smoke during cooking and the presence of garbage dumps near residences. Consequently, problems like persistent cough, persistent phlegm, wheezing, eye irritation, skin irritation, jaundice, asthma and dental caries have been observed to be more common in the industrial area [5–7]. Nepal falls in 173rd position in global air quality with particulate matter (PM) as the major air pollutant in Southeast Asia together with CO_2_, CO, SO_2_, NOx, and ozone. Burning of low grade coal is the major contributor to air pollution with PM 2.5 was 40, PM 10 was 120, TSP 41 was 230, SO_2_ was 70, NO_2_ was 80 in 24 hours and O_3_ was 157 in 8 hours [8].

To save high transportation expenses, many crushed stone operations are located near populous areas or along highways [9]. Stone crushing and associated activities contribute to particulate matter in the surrounding environment and exposure to dust can cause serious respiratory disease, skin, hearing, eye, and dyspnea like health problems including mortality [10, 11].

Exposure to petrochemical air emissions may be associated with increased rates of acute irritative symptoms, respiratory symptoms, and lower lung function than those living in other regions [11,12]. Particulate matter produced from brick kilns is harmful to human health as it causes respiratory diseases [13]. Brick kilns cause smoke-related respiratory discomfort at home and surroundings. The health status of the school children attending school close to the vicinity of the brick kiln was worse compared to the children attending school away from the brick kiln [14]. Brick kilns are the major single source of SO_2_ and SPM in the environment contributing to 60 percent of the emissions of Kathmandu valley [15].

Unplanned and unmanaged industrialization in the residential area leads to various health hazards and environmental degradation. In this study, the stone mines, crusher, and brick kiln were taken into consideration to assess the self-reported health effects and risk perception among residents living near industries in Godawari Municipality of Lalitpur, Nepal.

## Materials and Methods

### Study Setting

The research was centered around the communities residing within a 3 km radius of various industrial sites such as brick kilns, stone mines, and stone crushers within the Godawari Municipality. A suitable location for the study was carefully selected. The Godawari Municipality is situated in the Lalitpur district of Bagmati province in Nepal. Comprising 14 wards, the municipality hosts numerous brick kilns, stone mines, and stone crushers are available in wards 4, 5, 6, 7, 13, and 14.[Fig f1-eaht-38-4-e2023029]

In Kathmandu valley (Lalitpur, Bhaktapur and Kathmandu), 8% of the brick kilns of the country are operating but are emitting 28% of the total PM10 and 40% of carbon during winter season.

### Study Design

A descriptive cross-sectional study design was used to identify the self-reported health effects and risk perception of residents living near industries. The study period was from May 2019 to November 2019.

#### Sampling Procedure and Sample Size

Stone mine, stone crusher and brick kiln industries were selected purposively. A list of the participants who were living in the study sites was prepared. The below table shows the population around a 3 km radius of industries;

A simple random sampling technique was used for selecting the study sample. From each study sites, 45 eligible participants were selected for the study purpose. The sample size was calculated based on the study conducted on industrial air pollution in rural Kenya, which had a prevalence of perceived industrial pollution as posing considerable risk (80%) to the respondents [3]. With p=0.8, q=0.2, and z=1.96, we estimated a sample of at least 245 participants to be observed. After adding a 10% non-response rate, the final sample size was 270.[Table t1-eaht-38-4-e2023029]

#### Data Collection

The data was collected through face-to-face interviews, using a structured questionnaire which included: 1) sociodemographic information; 2) self-reported health status; and, 3) risk perception questionnaire. The questionnaire was developed in English following a literature review and was translated to Nepalese language to collect primary data and back-translated to English to confirm the accuracy of the translation. We first took permission to conduct data collection from each study site. Enough time was provided for each participant to understand each question. The collected data were checked for completeness before leaving the selected household.

#### Data Analysis

Before data entry, questionnaires were checked for completeness. All of the data was gathered only for the purpose of research. The Primarily collected data were verified for completeness and collected data were entered in Epi Data and were cleaned. The coded data were then exported to SPSS for processing and analysis.

For all continuous variables mean and standard deviation were calculated and for categorical variables frequency and percentage were calculated. A chi-square test was done to determine the association between the socio-demographic information, self-reported health status and risk perception. The association between the explanatory and dependent variables was assessed at a p-value of 0.005 and 95 % CI.

#### Ethical Considerations and Trustworthiness

Ethical approval was taken from the Nepal Health Research Council (3543). Similarly, written permission was taken from the Godawari Municipality for conduction of study. Informed verbal and written consent were obtained from the study participants before data collection. Before data collection, the respondents were thoroughly informed of the study's objective and other pertinent information. Participants were assured by the researcher about the confidentiality of the information, privacy, and anonymity of all respondents in the study. All ethical guidelines were followed.

## Results

The result is summarized in the table format. In some tables, the total does not equal to sample size due to nonresponse or where valid response of multiple responses is shown. In such cases, the number of respondents who responded to the question is indicated.

### Demographic and Social Characteristics of the participants

A description of the univariate analysis is given in Table 2 below.

Overall, 270 participants aged between 18-60 years living near industries of Godawari municipality were selected with 100% agreeing to participate in the study. Of the 270 respondents, 61.9 % were males higher than females (38.1 %) and more than half of the respondents (51.1 %) were aged between 40 to 60 years. Around one-third of respondents (36.7 %) had basic education and 10.3 % of respondents had university-level education. Most of the respondents follow (93.3 %) Hinduism. Nearly half of the participants (43.3 %) major occupation was agriculture and the least number of participants (11.9 %) were students. Nearly two-thirds (38.9 %) of respondents’ monthly family income was NPR 10,000-20,000.

### Self-reported disease

Among 270 participants, 110 participants experienced diseases during the last 12 months where 24 % experienced wheezing or whistling, 22 % experienced chest tightness, 21 % experienced shortness of breathe, 27 % experienced coughing, and 6 % had asthma.[Table t3-eaht-38-4-e2023029]

### Self-reported health status and stress

More than half (53.3 %) of participants responded to their self-assessed health status as good and the least number of them (1.1 %) responded as bad. Likewise, the majority of the respondents (65.6 %) responded to their sleep quality as good and half (51.1 %) of the participants responded that they were stressed by industrial activities sometimes, followed by two-thirds (36.3 %) who were always stressed by the ongoing industrial activities near the residence. Similarly, more than half (53.4 %) of participants were sad sometimes about the ongoing industrial activities near their residence along with 35.9 % who were always sad.[Table t4-eaht-38-4-e2023029]

### Risk perception

Nearly one-fourth (24.8 %) of participants responded that there is a high impact of industry on their original carrier. More than half of the participants (52.2 %) responded that industry had a high impact on the quality of air causing respiratory disease among residents. Similarly, more than half of the participants (51.1 %) responded that industry had a high impact on the quality of air causing several kinds of cancer among residents. Half of the participants (50.4 %) also responded that there is a high impact on the quality of air causing disease related to self-immunity disorder.[Table t5-eaht-38-4-e2023029]

### Psychological and nuisance effects from industrial development

In this study among 270 respondents, nearly half of the participants (48.5 %) responded that as a result of industrial development, they were considerably concerned about their health followed by 23 % strongly concerned, 20.4 % moderately concerned and 8.1 % were not concerned at all. Almost half (44.8 %) of respondents were considerably concerned about their future in that area and 27.4 % were strongly concerned, 20 % were moderately concerned and 8.1 % were not concerned at all. More than half (51.5 %) of the respondents disclosed that industries caused nuisances such as noise or smell sometimes in their area. Similarly, half (50.4 %) of the respondents responded that staying near the vicinity of industries irritated their eyes and nose sometimes.

### Impact, severity, perceived risk and benefits from the industry

Among the 270 respondents, more than half (54.8 %) of the respondents responded that there is a high probability that industries generate polluted air in that area and they will be impacted by air pollution. Half of the participants (51.9 %) were aware that there is a high severity of contaminated air in the area that affects humans. More than three-quarters of respondents (77.0 %) were not capable of protecting themselves from contaminated air in their area. The majority of the respondents (83.3 %) responded that industrial development in their area had not generated more income for their families.

### Association between industry and risk perception of participants

More than two-thirds of the participants (73.3 %) near the brick kiln, followed by stone mine (62.2 %) responded to the industry as a high risk. Furthermore, there was a significant association between industries and risk perception (p<0.001).[Table t6-eaht-38-4-e2023029]

### Association between self-reported health status and industries

One-third of participants living near brick kiln (38.9 %), stone mine (36.1 %), and stone crusher (25 %) assessed their sleep quality as average. Sleep quality was associated with industries (p<0.001). Participants living near industries like brick kilns (50 %), stone mines (14.3 %), and stone crushers (35.7 %) responded that they were always stressed by industrial activities near their residences. There was a significant association between stress by industrial activities near residence and industries (p<0.01). Likewise, there was also a significant association between industries and participant sadness about ongoing industrial activities near residence (p<0.01).[Table t7-eaht-38-4-e2023029]

## Discussion

The study insight into two major aspects, self-reported health status and risk perception of the participants living near the industries. The participants for the study were the residents living near different industries like brick kilns, stone mines, and stone crushers of Godawari municipality. However, they were aware of the industrial pollution, industrial hazards, and its effect on their lifestyle and health. Limitations of the study include a small sample size and a specific geographical area within 3 km near the industries. These data therefore cannot be generalized to other settings and other communities in Nepal. Self-reporting might lead to misinterpretation of diseases without physician’s report. Recall bias might have occurred due to the gap and duration from disease onset but the researcher had tried to reduce it.

Studies have shown that the presence of the industry in close proximity to residences, and industry has drawbacks from the point of view of public health. Consequently, problems like coughing, wheezing, eye irritation, skin irritation, and asthma have been observed to be more common in the industrial area. Also, the presence of all categories of self-reported health in the inhabitants of industrial areas compared with the reference community [5]. The Environment Protection Act of Nepal states that “No person shall create pollution in such a manner as to cause significant adverse impacts on the public life, public health and environment or do, or cause to be done, any act contrary to the standards determined by the Government of Nepal. The Ministry or Provincial Ministry can provide a pollution control certificate, as prescribed, to any industry that makes a significant contribution to the control of pollution [18]. However, there is a challenge in the effective implementation of the set environmental laws and regulations. The high prevalence of diseases particularly respiratory problems, eye irritations, hearing loss, cough, headache, vision defect, wheezing, skin irritation, chest pain, aliment problems, and hair loss reported problems among the population living in and around the neighborhood of Stone crusher industry [11] and similar health effects were expressed majority of respondents living near brick kilns in a study conducted in Kathmandu valley [14] and Bangladesh [19].

In our current study, approximately two-thirds of respondents were always stressed by the industrial activities near their residence, the results were similar to the study conducted in a petroleum refinery in Oak Vile, Ontario in Canada where residents’ sensitivity to the negative effects of the refinery on their health of their children suggests a psychological reaction to the environmental stress associated with perceived and actual refinery emissions [20]. About two-thirds of respondents always feel irritated in their eyes and nose staying near the vicinity of industries. The results are similar to the study carried out on the environmental health assessment of stone crushers around Jhansi, up, India [10] and Pammal, in suburban Chennai, the capital of Tamil Nadu State, India [9] which showed stone crushing and associated activities contribute to particulate matter in the surrounding environment.

More than half of the participants in the research were aware of the consequences of poor air quality, according to the findings. In order to limit their emissions in the environment, industries should implement current, efficient technologies. The majority of the participants discovered that they were unable to protect themselves against the industry's consequences. Also, the air quality monitoring system could be installed for continuous monitoring of pollutants in the study areas. Community-wide awareness programs should be conducted to encourage people to take certain safety precautions.

## Conclusions

More than half of the participants believed having industries near their homes poses a substantial risk. The findings revealed that the residents suffer from a variety of health issues, including wheezing or whistling, chest tightness, shortness of breathe, coughing, and asthma. Many of them were concerned about their future prospects in that community. Eco-friendly industries should be established in such a situation. However, there is a lack of attention given to enforcing and supervising the existing laws and regulations. The existing laws are less emphasized and there is less monitoring of industries. There should be strong coordination and collaboration between the responsible bodies like the Ministry of Environment, Ministry of Health and Population, Ministry of Local Development, Ministry of Physical Planning and Works and Municipalities. Meanwhile, to promote environmental awareness, regular training programs for residents on health and safety precautions should be arranged. The findings had far-reaching consequences for local-level planning and policy development, particularly in terms of gaining a better understanding of people's high-risk perceptions of industries near their homes.

## Figures and Tables

**Figure 1. f1-eaht-38-4-e2023029:**
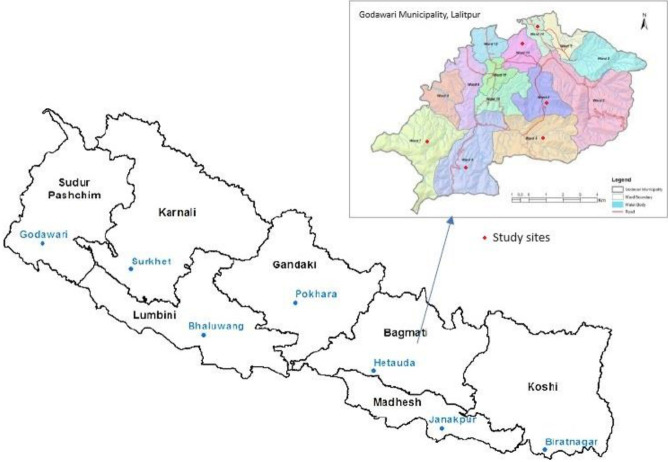
Map of the study site. Source: Godawari Municipality, 2016 [16]

**Table 1. t1-eaht-38-4-e2023029:** Total population around a 3 km radius of industries.

Ward No	Name of ward	Type of study site	Total population of around 3 km of industries
4	Badikhel	Brick Kilns	1835
5	Tikabhairav	Stone Crusher	523
6	Lele	Brick kilns	524
7	Devichaur	Stone Crusher	1047
13	Jharuwarashi	Brick Kilns	1048
14	Thaiba	Brick Kilns	1048

Source: population table of Godawari Municipality [17]

**Table 2.** Demographic and Social Characteristics of the Participants.

Variables	Frequency	% (n=270)
Sex		
Male	167	61.9
Female	103	38.1
Age in years		
20 or below	14	5.2
20-40 years	118	43.7
40-60 years	138	51.1
Education		
Illiterate	58	21.5
Basic education	99	36.7
Secondary	54	20.0
Higher Secondary	31	11.5
University level	28	10.3
Religion		
Hindu	252	93.3
Christian	10	3.7
Buddhist	8	3.0
Occupation		
Salary	35	13
Wages	46	17
Business	40	14.8
Agriculture	117	43.3
Student	32	11.9
Income		
Less than 5000	39	14.4
5000-10000	101	37.4
10000-20000	105	38.9
Above 20000	25	9.3

**Table 3. t3-eaht-38-4-e2023029:** Self-reported diseases.

Self-reported diseases	Frequency	Percentage (%)
Wheezing or whistling	26	24
Chest tightness	24	22
Shortness of breathe	23	21
Coughing	30	27
Asthma	7	6

**Table 4. t4-eaht-38-4-e2023029:** Self-reported health status and stress about industrial activities of the participant.

Variables	Frequency	% (n=270)
Self-assessed health status		
Very good	59	21.9
Good	144	53.3
Average	64	23.7
Bad	3	1.1
Self-assessed sleep quality		
Very good	54	20.0
Good	177	65.6
Average	36	13.3
Bad	3	1.1
Stressed by industrial activities near residence		
Always	98	36.3
Rarely	34	12.6
Sometimes	138	51.1
Sad about the ongoing industrial activities near residence		
Always	97	35.9
Rarely	29	10.7
Sometimes	144	53.4

**Table 5. t5-eaht-38-4-e2023029:** Lifestyle disruption, respiratory effect and physical health effect.

Variables	No Impact [n (%)]	Low Impact [n (%)]	High Impact [n (%)]
Impact of industry on original carrier	45 (16.7)	158 (58.5)	67 (24.8)
Impact of quality of air causing respiratory disease among residents	17 (6.3)	112 (41.5)	141 (52.2)
Impact of quality of air causing several kinds of cancer among residents	26 (9.6)	106 (39.3)	138 (51.1)
Impact of quality of air causing disease related to self-immunity disorder	15 (5.5)	119 (44.1)	136 (50.4)

(n=270)

**Table 6. t6-eaht-38-4-e2023029:** Association between industry and risk perception of participant.

Industry	Risk perception		ꭓ^2^	P
	Low risk n (%)	High risk n (%)		
Brick kiln	24 (26.7 %)	66 (73.3 %)		
Stone mine	67 (74.4 %)	23 (25.6 %)	45.2	P < 0.001
Stone crusher	34 (37.8 %)	56 (62.2 %)		
Total	125 (46.3 %)	145 (53.7 %)		

(n=270)

**Table 7. t7-eaht-38-4-e2023029:** Association between self-reported health status and industries.

Variables	Industry			ꭓ^2^	P
	Brick kiln n (%)	Stone mine n (%)	Stone crusher n (%)		
Sleep quality					
Very good	33 (61.1%)	9 (16.7%)	12 (22.2%)		
Good	42 (23.7%)	68 (38.4%)	67 (37.9%)	29.5	P < 0.001
Average	14 (38.9%)	13 (36.1%)	9 (25.0%)		
Bad	1 (33.3%)	0	2 (66.7%)		
Stress by industrial activities near residence					
Always	49 (50.0%)	14 (14.3%)	35 (35.7%)		P < 0.001
Rarely	10 (29.4%)	12 (35.3%)	12 (35.3%)	31.3	
Sometimes	31 (22.5%)	64 (46.3%)	43 (31.2%)		
Sad about ongoing industrial activities near residence					
Always	49 (50.5%)	14 (14.4%)	34 (35.1%)		P < 0.001
Rarely	8 (27.6%)	6 (20.7%)	15 (51.7%)	39.4	
Sometimes	33 (22.9%)	70 (48.6%)	41 (28.5%)		

(n=270)

## References

[1] Zhao J, Yiengprugsawan V, Seubsman S, Kelly M, Bain C, Sleigh A (2014). Self-reported health and subsequent mortality: an analysis of 767 deaths from a large Thai cohort study. BMC Public Health.

[2] Janmaimool P, Watanabe T (2014). Evaluating determinants of environmental risk perception for risk management in contaminated sites. Int J Environ Res Public Health.

[3] Omanga E, Ulmer L, Berhane Z, Gatari M (2014). Industrial air pollution in rural Kenya: community awareness, risk perception and associations between risk variables. BMC Public Health.

[4] https://www.who.int/news/item/13-06-2007-new-country-by-country-datashow-in-detail-the-impact-of-environmental-factors-on-health.

[5] Saha A, Kulkarni P, Saiyed H (2007). Living environment and self assessed morbidity: a questionnaire-based survey. BMC Public Health.

[6] Legator MS, Singleton CR, Morris DL, Philips DL (1998). The health effects of living near cement kilns; a symptom survey in Midlothian, Texas. Toxicol Ind Health.

[7] Wilson D, Takahashi K, Pan G, Chan CC, Zhang S, Feng Y (2008). Respiratory symptoms among residents of a heavyindustry province in China: prevalence and risk factors. Respir Med.

[8] http://hdl.handle.net/10986/33727.

[9] Sivacoumar R, Jayabalou R, Swarnalatha S, Balakrishnan K (2006). Particulate matter from stone crushing industry: Size distribution and health effects. Journal of Environmental Engineering.

[10] Sheikh A, Rana SVS, Pal A (2011). Environmental health assessment of stone crushers in and around Jhansi, U. P., India. Journal of Ecophysiology and Occupational Health.

[11] Ganesh S, Singh MM (2019). Impact of stone crusher on ambient air quality and human health Jhansi region in Bundelkhand U.P. International Journal of Scientific & Technology Research.

[12] Wichmann FA, Müller A, Busi LE, Cianni N, Massolo L, Schlink U (2009). Increased asthma and respiratory symptoms in children exposed to petrochemical pollution. J Allergy Clin Immunol.

[13] Yang CY, Wang JD, Chan CC, Chen PC, Huang JS, Cheng MF (1997). Respiratory and irritant health effects of a population living in a petrochemical-polluted area in Taiwan. Environ Res.

[14] Joshi SK, Dudani I (2008). Environmental health effects of brick kilns in Kathmandu valley. Kathmandu Univ Med J (KUMJ).

[16] https://www.nepalarchives.com/content/godawari-municipality-lalitpur-profile/.

[17] https://www.citypopulation.de/en/nepal/kathmanduvalley/.

[18] https://lawcommission.gov.np/np/.

[19] Tusher TR, Ashraf Z, Akter S (2019). Health effects of brick kiln operations: A study on largest brick kiln cluster in Bangladesh. South East Asia Journal of Public Health.

[20] Luginaah IN, Taylor SM, Elliott SJ, Eyles JD (2002). Community reappraisal of the perceived health effects of a petroleum refinery. Soc Sci Med.

